# Association between milk urea nitrogen and first service conception in smallholder dairy farms under heat and humidity stress

**DOI:** 10.14202/vetworld.2018.1604-1608

**Published:** 2018-11-22

**Authors:** Suppada Kananub, John A. VanLeeuwen, Pipat Arunvipas

**Affiliations:** 1 Department of Large Animals and Wildlife Clinical Sciences, Faculty of Veterinary Medicine, Kasetsart University, Bangkok, Thailand; 2 Department of Health Management, Atlantic Veterinary College, University of Prince Edward Island, Charlottetown, Canada

**Keywords:** first service conception, milk urea nitrogen, smallholder farm

## Abstract

**Aim:**

The study was to evaluate the relationship between the first service conception (FSC) and milk urea nitrogen (MUN) in smallholder dairy farms under heat and humidity stress.

**Materials and Methods:**

Dairy cows from 43 dairy farms giving birth between November 2014 and April 2015 (n=295) contributed to the study. All cows were sampled monthly to measure milk compositions, and we collected additional farm data from farmers through a questionnaire. The first service during the first 120 days of lactation was the outcome of interest in this study. Multivariable logistic regression determined significant associations with FSC.

**Results:**

The overall FSC was 22% and the mean MUN concentration was 11.55 mg/dl. The final FSC model included MUN concentration, the season of breeding, and protein energy ratio (PE ratio) in the diet. The odds of FSC were reduced by approximately 10% for each mg/dl higher MUN on the day of the milk sample that was nearest to the artificial insemination (AI) day. The odds of FSC were nearly 3 times higher when the first insemination occurred in winter compared to summer first services. Taking into account the nutritional factors, the odds of FSC were nearly 70% higher with an increase in PE ratio of 10 g of crude protein/Mcal from the mean of 35.90 g.

**Conclusion:**

This study of smallholder dairy farmers in the hot and humid climate of Thailand confirmed that season, nutritional management, and MUN concentration were associated with FSC. MUN appears to be a useful indicator to monitor the effects of diet on reproductive performance from this study.

## Introduction

The formation of urea in the liver occurs to reduce tissue toxicity of ammonia above amounts utilized by ruminal microbes [1,2]. Urea is transported from the liver through blood circulation to other body parts and diffuses into other tissues, for instance, the udder and uterus [3]. Milk urea nitrogen (MUN) can be advantageously used for evaluating urea levels in tissue because the MUN level highly correlates well with blood urea nitrogen level and is easily obtained through non-invasive techniques [2], often through monthly milk testing programs. Urea, diffusing through the reproductive tract, can impair reproductive functions [4]. First service conception (FSC) has been negatively correlated with MUN in temperate climates [5,6]. In areas with hot and humid summers but moderate winters, such as Florida, the effect of MUN on fertility appears to be modified by weather; cows exposed to high MUN in the summer had lower conception but not in the winter [7].

Therefore, MUN can be used as a predictor for nutritional management impacting reproductive success [4,5]. However, few studies correlating MUN and reproductive success have been performed in countries with year-round hot and humid environments, and no studies have examined this relationship in small-scale dairy farms, which are the main kind of farms in Thailand and other countries in the global south. The average daily temperature (standard deviation [SD]) in Thailand never goes below 27°C (2.57°C), and the average humidity never goes below 73% [8,9].

The objective of this study was to investigate the association between MUN and FSC in smallholder farms existing in hot and humid areas all year round.

## Materials and Methods

### Ethical approval

This study obtained the permission of the Animal Ethics Committee of Laboratory Animals, Veterinary Technology, Kasetsart University (59-VET-031).

### Farm and cow sample

The sampling frame of the study included the dairy farms which were serviced by Kasetsart University. Inclusion criteria for farm selection were as follows: (1) reproductive data were recorded routinely, (2) <30 milking cows, (3) voluntary waiting period (VWP) was set at 50 days, and (4) consent to collect milk samples from all lactating cows monthly. With the VWP being approximately 2 months, cows typically have another 2 months after the VWP for their first service, which was assumed to be three estrus cycles. Not all cows cycle during the VWP and therefore such anestrus cows do not have the first service shortly after the VWP but they do cycle once the negative-energy balance is rectified when their dry matter intake finally reaches its peak at around 90 days after parturition. Reducing this window of days for FSC would lead to observations dropped, limiting the power of the study to find relationships between FSC and MUN. Therefore, we think that breeding within the first 120 days was acceptable for the study of first service conception.

Farms from two dairy cooperatives, Nong-Pho and Ta-Muang, agreed to participate in the study. Those two cooperatives are centers for collecting milk, where they also normally provide artificial insemination (AI) services and sell concentrates for cow diets. There are differences between the two centers regarding the availability of forage resources that could affect the cows’ diets in their areas. For example, silages are more eligible in Nong-Pho than in Ta-Muang, and concentrates provided to farmers by the cooperatives are different. All cows calving on these farms between November 2014 and April 2015 were eligible to be followed post-partum for breeding.

### Data collection

Nutritional, farm management, and reproductive data were obtained from farmers through a brief questionnaire. The parts of the questionnaire were as follows: (1) barn characteristics, (2) herd characteristics, (3) breeding practices, and (4) feeding practices. There were both closed- and open-ended questions. About 10 min was needed to complete the questionnaire. The questionnaire was pretested on 10 farmers for clarity. The project budget allowed for one dietary assessment per farm at the midpoint of the study and these nutrient data were combined with data on feeding practices to estimate nutrient intakes.

All eligible cows had milk samples collected monthly. Milk compositions were measured on these samples by a CombiFoss^®^ machine at Kasetsart University. Parameters measured included MUN concentration, the percentage of milk total solids, lactose, fat and protein, and somatic cell count (SCC).

### Data management and analyses

For our descriptive statistical analyses, proportions and means (SD) were calculated for the data. For our inferential statistical analyses, conception at first service was the dependent outcome of interest. MUN was the main independent variable of interest, and for this variable, the MUN concentration on the day nearest to the AI was selected for identifying the relationship between MUN and FSC. Logistic regression was used for the analysis that examined not only MUN as a factor of FSC but also other non-nutritional parameters (i.e. milk constituents, SCC, breeding season, cooperative center, and farm management parameters) and nutritional parameters (i.e., total dietary protein, total dietary energy, and protein energy ratio [PE ratio]) linked to the first service data for each cow. We recognize that not all parameters were related to the objectives but, with the data available, we wanted to confirm that these variables were not confounders of other relationships we were finding.

Lowess smoothing curve and fractional polynomials were used to determine the best form of the continuous independent variables in the model (i.e., curvilinear, linear, or categorical), along with biological plausibility. The analyses started with unconditional univariate logistic regression, and variables which met a significance cutoff level of p<0.2 were used for the next steps. Multivariable logistic regression, with farm random effect to control for cows clustered within farms, was conducted, with the forward manual stepwise addition of the most significant variables that contributed to the final model. Interactions and confounding factors were also tested for significance [10]. A cutoff of p=0.05 was used for determining the significance of each parameter. Bonferroni comparison test was utilized for evaluating the pairs of difference. The Hosmer–Lemeshow test was used for evaluating goodness-of-fit of the model. The analyses were performed in the STATA statistical software package (version 13.0, Stata Corp., College Station, TX).

## Results

There were 46 farms meeting the eligibility criteria for the study. However, there were 12 cows from three farms on which the tested feed ration was not representative of normal feeding; therefore, those cows and farms were excluded from the analyses. Furthermore, as this study was particularly interested in FSC, which should normally happen during the first 120 days of lactation, 174 observations were dropped because they were not bred or the first service occurred after 120 days of lactation. Therefore, the analyses were performed on data from 295 cows from 43 farms.

### Descriptive statistics

Descriptive statistics of the farm, nutrition, and non-nutrition variables are shown in Table-1, along with a stated p-value from the unconditional univariable logistic regression with FSC. Herd size was <20 milking cows in >50% of farms. Farms in this study were nearly 60% tie-stall. Over 60% of studied farms had a concrete floor only for resting areas, while nearly 40% were concrete covered by a rubber mat. The number of samples from Nong-Pho center (n=191) was 2 times (Table-1) that of Ta-Muang center (n=104).

**Table-1:** Descriptive statistics of parameters analyzed by unconditional univariable logistic regression for associations with first service conception (p-value), among 295 cows on 43 smallholder dairy farms in Thailand in 2014-2015.

Parameters	Mean±SD	%	p-value
Farm parameters herd size	18.60±6.99	-	0.88
Barn type			
Tie stall	-	58.65	0.35
Free stall	-	41.35	-
Barn floor			
Concrete	-	63.14	0.20
Rubber	-	36.86	-
Cooperative			
Nong Pho	-	66.35	0.03
Ta-Muang	-	33.65	-
Nutritional parameters			
CP (kg/day)	2.79±0.93	-	0.19
Gross energy (Mcal/day)	89.06±13.32	-	0.59
PE ratio (g of CP/Mcal)	35.90±7.60	-	0.03
Non-nutritional parameters			
Milk fat (%)	4.00±3.84	-	0.86
Milk protein (%)	2.74±0.30	-	0.64
Milk lactose (%)	4.71±0.33	-	0.53
Total solids (%)	12.13±3.58	-	0.83
SCC (×103 cells/ml)	80.64±6.96	-	0.76
MUN	11.55±3.76	-	0.01
Lactation No.	2.89±1.99	-	0.08
Breeding season			
Summer	-	19.87	0.05
Rainy	-	38.78	-
Winter	-	41.35	-

a Back-transformation of average natural-logarithm of somatic cells. PE=Protein energy, SCC=Somatic cell count, MUN=Milk urea nitrogen, SD=Standard deviation

Two main nutrition parameters of interest were crude protein (CP) and gross energy, with averages of 2.79 kg and 89.06 Mcal per day of CP and gross energy, respectively. These parameters led to a low average PE ratio of 35.90 g of CP/Mcal. For the non-nutritional factors, the variation in milk fat of the cows was quite high; the average (SD) of milk fat percentage was 4% (3.84%). Interestingly, the average milk protein percentage was lower than 3%, whereas average milk lactose was higher at 4.7%, with total solids of 12.1%. The average of SCC as a geometric mean was nearly 80,000 cells/ml after back-transformation. It was suspected that SCC could have a negative effect on FSC, but we did not find a significant association between FSC and SCC. The MUN average (SD) was 11.55 mg/dl (3.76 mg/dl). The average lactation number of studied cows was almost three. Nearly 20% of the first services occurred during the summer season, with nearly 40% of first services occurring during each of the rainy and winter seasons. Cooperative, CP, PE ratio, MUN, lactation number, and breeding season met the significance criteria of p<0.20 and were eligible for the multivariable logistic regression modeling.

### Analytical statistics

**Figure-1:**
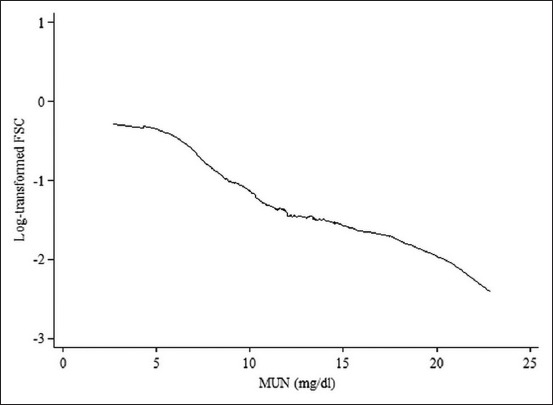
Lowess smoothing curve is testing the linearity of the relationship between milk urea nitrogen concentration and first service conception among 295 cows on 43 smallholder dairy farms in Thailand in 2014-2015.

Table-2 presents the odds ratios of three significant parameters, MUN, breeding season, and PE ratio, associated with FSC in the final multivariate logistic regression. In Figure-1, the Lowess smoothing graph presents the inverse linear association between log odds of FSC and MUN. The odds of FSC were reduced by approximately 10% for each 1 mg/dl increase in MUN concentration on the day nearest to the AI day.

**Table-2:** Parameters associated with conception at first service from the final multivariable logistic regression among 295 cows on 43 smallholder dairy farms in Thailand in 2014-2015.

Parameters	MUN as categorical variable		

	OR	SE	p-value
MUN	0.88	0.04	0.002
Breeding season			
Summer	Reference		
Rainy	1.47	0.65	0.40
Winter	2.93	1.25	0.01
PE ratio (10 g of CP/Mcal)	1.71	0.36	0.01
Constant	0.14	0.05	<0.0001

OR=Odds ratio, SE=Standard error, MUN=Milk urea nitrogen, PE=Protein energy, CP: Crude protein

**Figure-2:**
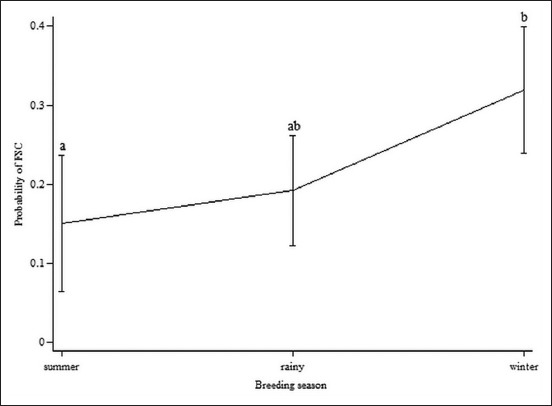
Probabilities of first service conception corresponding to breeding seasons among 295 cows on 43 smallholder farms in Thailand in 2014-2015.

For the breeding season, the probabilities of FSC increased from summer to rainy and winter seasons (Figure-2). However, only FSC of cows bred in the summer significantly differed from the winter season (p<0.05). The odds of FSC in cows bred in the winter were nearly three times the odd of FSC in cows bred in the summer season.

The Lowess smoothing graph showed a linear relationship between PE ratio and FSC. As the PE ratio was low, for ease of interpretation of the PE ratio, the mean was set to be the reference value of the PE ratio, and therefore, the odds ratio for the PE ratio variable corresponded to an increase of 10 g of CP/Mcal from the mean, instead of 1 g of CP/Mcal (Table-1), since PE ratios were divided by 10 before inclusion into the final model. The National Research Council has recommended that the PE ratio in small breed cows producing 15 kg of milk should be 90 g of CP/Mcal [1]. The level of PE ratio in the diet was positively associated with FSC. Cows that obtained 10 g of CP/Mcal above the mean of 35.90 g of CP/Mcal had increased odds of FSC by 70%.

When examining the final model, the analyses did not reveal any significant interactions or confounding factors or any problems with residual and influential data. Hosmer–Lemeshow goodness-of-fit test is the measure used for the evaluation of model fit because of the small number of data points per covariate. Data were divided into 10 covariates, the typical number for a Hosmer–Lemeshow test. The p-value of the test was 0.4, indicating that the fitted model was suitable.

## Discussion

In our study of smallholder dairy cows in the hot and humid climate of Thailand, we demonstrated the detrimental influence on their reproductive performance when MUN increased, even while controlling for season and PE ratio in the final model. This finding agrees with the studies in other contexts [5-7] but not with all studies [11,12]. Previous findings have shown the deterioration of oocyte and sperm viability, the uterine environment, and reproductive hormones associated with elevated MUN [4,13]. Other mechanisms of reproductive impairment proposed include an alteration gene affecting immune function, or a change in lipid metabolism, and/or uterine involution [14]. However, the nutritional management of feeds is important; pregnancy rates have been shown not to be affected by increased amounts of CP in the diet if the dietary protein is balanced with energy in the diet [12].

Each 10 g increase in CP/Mcal for the PE ratio from the study mean of 35.90 g was significantly associated with nearly a 70% increase in FSC. Elsewhere, a positive relationship between the timing of first ovulation post-calving and the PE ratio was documented [15]. The National Research Council recommends that the PE ratio in small breed cows producing 15 kg of milk should be 90 g of CP/Mcal [1]. This ratio indicates the desired protein intake relative to each Mcal unit of energy intake for efficient rumen microbial activity. Since our mean of PE ratio was quite low, it is not surprising that the conception at first service was higher with a higher PE ratio. We deduce that there was low protein intake in some farms from our low mean PE ratio and that protein deficiency, especially in early lactation, had an impact on the reproductive efficiency [16,17]. Prolonged days open, increased number of services per conception, and decreased pregnancy rate have been reported problems associated with inadequate protein intake elsewhere [16,18].

Season of first breeding was also significantly related to the conception at first breeding in our study; the highest FSC took place in the winter, and the lowest FSC was in the summer, agreeing with other studies [19]. Elsewhere, the proposed effects of high temperature on impaired fertility were reported to be occurring directly and indirectly, as described below [20]. According to the known relationship between body temperature and environmental temperature, hot season disadvantaged follicular development and fetal implantation [20,21]. Moreover, summer months have been shown to lead to reproductive hormone disturbance, deteriorated oocyte development, and early fetal death [19]. In addition to these direct effects of high temperature on reproduction, summer months have also been shown to indirectly influence reproduction by low protein and digestibility in the pasture and decreased feed intake, resulting in a negative change in energy balance [18,22]. Our results slightly differed from these seasonal effects on FSC during 4 months after calving; there was no significant difference in FSC between the rainy season and summer season in our data. During the rainy season, there were likely lots of fresh pasture being available, enhancing reproductive hormone synthesis [6,18]. With the direct and indirect impacts of the season on FSC, it was important to control for the season in the multivariable model to ensure that it was not confounding the observed relationship between MUN and FSC.

There were a couple of limitations of the findings in our study. While we had milk composition data and farm management data, we did not have monthly milk yield data (farmers did not record this information) nor did we have monthly nutritional data (the budget allowed for one dietary assessment at the midpoint of the study). These other data would have been useful for the analysis, particularly if the data on monthly diet were available and examined at the actual time point of breeding. As another study limitation, the statistical analyses were done with 295 cows, since 174 cows were not included because they were not bred by 120 days after calving. Cows not being bred before 120 days could be confounded by other factors affecting reproductive performance (e.g., endometritis), and therefore, the results of these analyses are restricted to cows <120 days in milk.

## Conclusion

The factor of interest, MUN was significantly negatively associated with FSC; when MUN concentration on the day nearest to AI day was increased by 1 mg/dl, there was a decrease of about 10% to the odds of FSC. With the study in a hot and humid climate area, there was a significant seasonal effect on the reproductive performance; FSC was highest in winter breeding, while the breeding success in the rainy season was slightly but not significantly higher than the summer season. In the study conditions of low PE ratios, an increase in the PE ratio was also associated with an increased FSC. Therefore, PE ratio and MUN were found to be important factors to monitor the success of reproductive management.

## Authors’ Contributions

JAV and PA are main supervisors, giving suggestions in all steps of the study. SK was the Ph.D. student on the study and had the responsibilities for sample collection, data analyses, and the manuscript writing. All authors read and approved the final manuscript.
